# Item response theory-based psychometric analysis of the Short Warwick-Edinburgh Mental Well-Being Scale (SWEMWBS) among adolescents in the UK

**DOI:** 10.1186/s12955-023-02192-0

**Published:** 2023-09-29

**Authors:** Radka Hanzlová, Peter Lynn

**Affiliations:** 1 Institute of Sociology of the Czech Academy of Sciences, Prague, Czech Republic; 2 University of Essex, Colchester, UK

**Keywords:** SWEMWBS, IRT, Psychometric properties, Mental well-being, Adolescence, UK, Understanding society

## Abstract

**Background:**

Middle and late adolescence is the period in a person's life that is most vulnerable to mental health problems. To enable an evidence base that can support policies to prevent such problems, it is crucial to have good quality, reliable, and accurate measurement tools for mental well-being. One of them is the Short Warwick-Edinburgh Mental Well-Being Scale (SWEMWBS). This study aimed to test the psychometric properties of the SWEMWBS on a large sample of adolescents aged 16 to 19 from the United Kingdom (UK) (*N* = 8,090). Data were from four waves of the longitudinal panel study *Understanding Society*.

**Methods:**

The analysis was conducted using Item Response Theory (IRT), which is the most appropriate method for testing psychometric properties. The Graded Response Model (GRM) was applied to the data. The reliability and criterion validity of the SWEMWBS were also examined.

**Results:**

The presented results confirm the very good psychometric properties of the SWEMWBS amongst adolescents aged 16 to 19 years. The assumptions for the use (unidimensionality, local non-independence, monotonicity) of IRT were met. The results of GRM showed very high discriminant power for all items. The five-category response scale performed optimally; however, differences were found between points on the response scale both between and within items. In general, the scale as a whole showed very good functioning, but particularly in the negative values of mental well-being.

**Conclusions:**

The SWEMWBS was confirmed as a concise, reliable, and valid instrument for measuring mental well-being among older UK adolescents.

**Supplementary Information:**

The online version contains supplementary material available at 10.1186/s12955-023-02192-0.

## Background

Adolescence is a developmental period in a person’s life lasting from approximately 10 to 19 years [1]. It is the stage in which an individual matures and undergoes a great deal of physical, cognitive, emotional, and psychological development and change. Early adolescence (ages 10 to 13) is mainly a period of physical change (e.g., height growth, change of body structure), while middle and late adolescence, approximately 16 to 19 years of age, is a period of cognitive development, emotional changes, perception and formation of personality, identity, and independence. The latter is a challenging period for the individual and prone to mental health difficulties [2]. This is particularly evident in recent years when mental health problems among adolescents and young people have increased all over the world. According to the World Health Organization (WHO), one in seven 10 to 19 year-olds globally suffer from a mental disorder [3]. In the United Kingdom (UK), the rate of mental health problems in adolescents aged 17 to 19 was 25.7% in 2022, which is a rise from just over 15% in 2017 [4].

It is, therefore, absolutely crucial to use accurate and validated tools for measuring mental health in this target group in order to be able to monitor trends and to provide analysis that can be used to implement various interventions for promoting adolescents’ mental health. There are many tools available to measure mental health (e.g., [5–7]). However, these have mostly been tested on respondents from the adult population or adolescents aged up to 15 years or a small specific sample through Classical Test Theory (CTT). One of the most widely used measures of mental well-being is the 14-item Warwick-Edinburgh Mental Well-Being Scale (WEMWBS) [8], and the related short seven-item version (SWEMWBS), which is, according to the authors, preferable to the full version [9]. This scale represents mental well-being as on a continuum, with mental problems or mental illness at one end and mental health at the other [8].

This paper aims to test the psychometric properties of SWEMWBS on a large sample of UK adolescents aged 16 to 19 years through Item Response Theory (IRT). Although validated studies of this scale in English (e.g., [10–12]) as well as in other languages (e.g., [13]) exist, none of them specifically targeted this age group nor applied IRT. The biggest advantage of IRT is that it provides information about the functioning of the scale as a whole (scale level) as well as each item within the scale (item level), which can inform the creation of a concise and psychometrically valid measure [14]. This technique can also detect the functioning of the response scale and identify the respondents’ response style.

## Methods

### Sample and data

Analysis was based on data collected by a combination of CAPI (Computer Assisted Personal Interviewing) and online self-completion questionnaire methods on *Understanding Society*, the largest longitudinal panel study in the UK started in 2009 (www.understandingsociety.ac.uk). Because the target group is narrow (adolescents aged 16 to 19 years) and the measurement instrument (SWEMWBS) was only used in four waves (Wave 1 (2009–2011), Wave 4 (2012–2014), Wave 7 (2015–2017), and Wave 10 (2018–2020)), the individual waves were treated as cross-sectional and were pooled to increase statistical power.^1^ All data were weighted with appropriate weights as recommended (cross-sectional weights from each wave pooled into one). Only respondents who answered all SWEMWBS items were included in the analysis. The final research sample (*N*) comprised 8,090 respondents aged 16 to 19 (mean age 17.3 years), of which 4,226 were male (51.7%) and 3,950 female (48.3%).

### Measures

#### The Short Warwick-Edinburgh Mental Well-Being Scale (SWEMWBS)^2^

The SWEMWBS developed from the original 14-item WEMWBS by Stewart-Brown et al. [9] comprises seven items rated on a 5-point Likert scale: *none of the time* (1), *rarely* (2), *some of the time* (3), *often* (4), *all the time* (5). All items are phrased positively worded and cover both aspects of mental well-being – feeling good and functioning well. The total score ranges from 7 to 35 (the higher score, the higher level of mental well-being). However, the raw scores need to be transformed into metric scores [9]. The exact wording of the items is shown in Table 1.

**Table 1:** Descriptive statistics for SWEMWBS 7 items (*N* = 8,090)

	***M***	***SD***	**Skewness**	**Kurtosis**	***r***_**it**_
I’ve been					
Item 1: feeling optimistic about the future.	3.37	1.00	–⁠3.35	–⁠0.26	.50
Item 2: feeling useful.	3.35	0.94	–⁠0.40	–⁠0.05	.62
Item 3: feeling relaxed.	3.38	0.96	–⁠0.27	–⁠0.40	.62
Item 4: dealing with problems well.	3.47	0.92	–⁠0.43	–⁠0.06	.71
Item 5: thinking clearly.	3.55	0.92	–⁠0.44	–⁠0.10	.71
Item 6: feeling close to other people.	3.66	0.98	–⁠0.50	–⁠0.18	.60
Item 7: able to make up my own mind about things.	3.91	0.89	–⁠0.69	0.33	.62

*M* mean, *SD* standard deviation, *r*_it_ corrected-item total correlation

#### General Health Questionnaire (GHQ)

The GHQ is a 12-item instrument for measuring psychological distress [15]. Respondents are asked questions about their feelings over the last few weeks, which they answer using a 4-point scale (the higher score, the worse mental health). Example of items: “Have you recently (a) lost much sleep over worry, (b) felt constantly under strain, or (c) been able to enjoy normal day-to-day activities.”

#### Overall life satisfaction

A single question was used to measure overall life satisfaction, which respondents answered using a 7-point Likert scale ranging from *completely dissatisfied* (1) to *completely satisfied* (7). The wording was: “Here are some questions about how you feel about your life. Please choose the number which you feel best describes how dissatisfied or satisfied you are with the following aspects of your current situation. Your life overall.”

#### General health

Subjective general health was measured with a single item asking respondents, “In general, would you say your health is… *excellent* (1), *very good* (2), *good* (3), *fair* (4), or *poor* (5)?”.

### Statistical methods

Data preparation and all preliminary analyses, including descriptive statistics, reliability, validity, and unidimensionality testing, were conducted using the statistical software SPSS 27. IRT analysis was carried out in STATA 17 and R using package *mirt*. Since the items are polytomous and ordered, the two most commonly used models were applied to the data and compared, namely the General Partial Credit Model (GPCM) [16] and the Graded Response Model (GRM) [17]. In both models, one discriminant parameter or slope (*a*) and four difficulty or threshold parameters (*b*) (number of response scale points minus one) are estimated for each item. The discriminant parameter (*a*) indicates how well or poorly an item discriminates between respondents with different levels of the latent trait (referred to as theta, *θ*) and also how strongly the item relates to *θ*. The values of parameter *a* can theoretically range from –∞ to + ∞ but most often range from 0 to 2, with higher values being desirable [18]. The interpretation of difficulty or threshold parameters (*b*) differs. In GPCM *b* denotes the value of the latent variable required to move between two adjacent categories on the response scale, whereas in GRM *b* refers to the 50% probability that the respondent will choose a given category on the response scale or higher. The values of the *b* parameter most often range from –2 to 2 [19].

The evaluation was also based on graphs, which represent another advantage of IRT over CTT. The graphs in question were Item characteristics curves (ICCs), Category characteristic curves (CCCs), Item information functions (IIFs), and Test information function (TIF).

## Results

### Descriptives

Table 1 shows the descriptive statistics and the results of testing normality distribution. The mean item scores ranged from 3.35 (item 2) to 3.91 (item 7). The skewness and kurtosis values were low, indicating no evidence of a difference from the normal distribution. All items are very strongly related to the scale since the corrected-item total correlation was 0.50 or higher.

### Unidimensionality, monotonicity, and local independence

Three assumptions must be tested before IRT can be applied [18]. First, unidimensionality was tested through principal component analysis (PCA), which clearly extracted one factor with eigenvalue 3.82, explaining 54.57% of the variance (for more detail, see Table A1 and Fig. A1 in Additional file 1). Second, local independence was tested by checking the residual correlation between pairs of items using the Yen Q3 test [20]. Chen and Thissen [21] suggest that local independence is questioned when the correlation is greater than 0.20. The results showed that the correlation between several items was slightly above the threshold of 0.20, with a maximum of 0.26 (see Table A2 in the Additional file 1). Given that the main recommendation to prevent local dependence is good questionnaire instrument development and positive wording of the items in the scale, which is met in the case of the *Understanding Society* survey and SWEMWBS, the items can be considered locally independent. Last, all items were monotonically increasing. This means that choosing a higher category on the response scale indicated a higher level of mental well-being. Therefore, it can be concluded that all three assumptions (unidimensionality, monotonicity, and local independence) for IRT analysis are met.

### IRT analysis

At first, the model fit of the GPCM and GRM results was compared. Three fit indices were used for evaluation: *Log-likelihood*, *Bayesian information criterion* (BIC), and *Akaike information criterion* (AIC). The results showed that GRM was preferable since *Log-likelihood* was higher (GRM = –64629.06 vs. GPCM = –65555.10), and AIC (GRM = 129328.10 vs. GPCM = 131180.20) and BIC (GRM = 129573.40 vs. GPCM = 121425.50) lower. GRM was therefore applied for the next steps of IRT analysis.

**Fig. 1:**
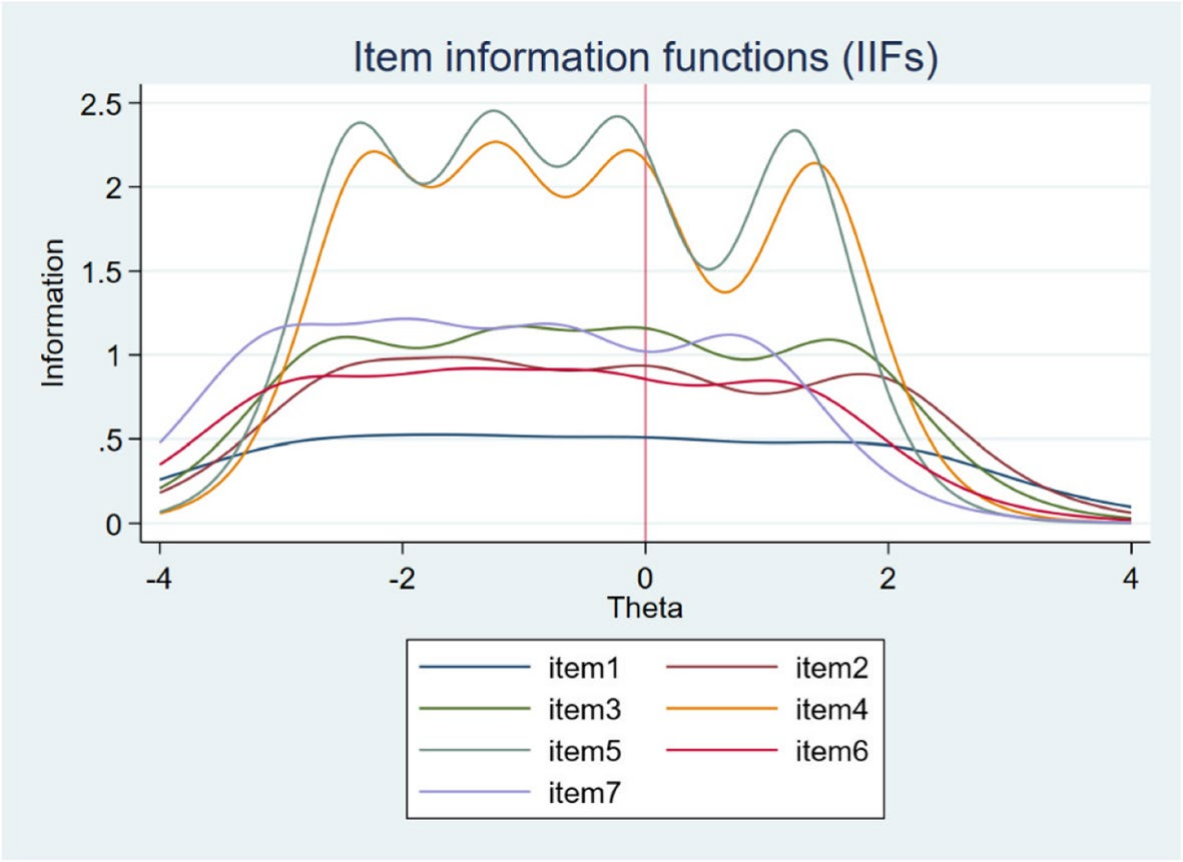
Item information functions (IIFs) for seven items of the SWEMWBS with a vertical line at *θ* = 0

The discrimination parameter (*a*) and threshold parameters (*b*) calculated from GRM appear in Table 2. The results showed that all items discriminate very well since the parameter *a* can be considered as “high” for item 1 and “very high” for other items according to guidelines by Baker [19]. The highest value of the parameter was 3.04 (item 5), and the lowest was 1.33 (item 1). These results are clearly illustrated by the IIFs (Fig. 1), which show that the least discriminating item 1 is placed lowest and, in terms of shape, is the most flat. By contrast, the items with the largest value of parameter *a* contain more information and are characterized by variability in the shape of the curve. The values of the discrimination parameter correspond with the informative contribution, i.e., the most discriminative items are also the most informative and vice versa.

**Table 2:** Discrimination and thresholds parameters for SWEMWBS

	Discrimination parameter	Difficulty parameters for each threshold			
	*a*	*b1*	*b2*	*b3*	*b4*
Item 1	1.33	− 2.85	− 1.50	0.05	1.91
Item 2	1.85	− 2.48	− 1.36	0.06	1.86
Item 3	2.05	− 2.58	− 1.16	0.03	1.60
Item 4	2.91	− 2.30	− 1.22	− 0.10	1.41
Item 5	3.04	− 2.39	− 1.26	− 0.19	1.25
Item 6	1.78	− 2.91	− 1.56	− 0.38	1.17
Item 7	2.07	− 3.07	− 1.93	− 0.71	0.79

Four difficulty parameters were estimated for each SWEMWBS item, 28 in total. Most (18) have a negative value, and the remaining 10 have a positive value, indicating that the scale is better able to measure and discriminate between respondents with a negative value for latent trait, i.e. low mental well-being. The values of parameter *b* ranged from − 3.07 (item 7) to 1.91 (item 1). The interpretation of the values themselves can be illustrated by the specific example of item 1, for which the parameter value *b1* =  − 2.85 means that a respondent with a latent trait level (*θ* =  − 2.85) has a 50% chance of answering item 1 with category 2 or higher; a respondent with *θ* =  − 1.50 has a 50% chance of answering with categories 3 to 5 rather than categories 1 or 2; a respondent with *θ* = 0.05 has a 50% chance of answering with categories 4 to 5 rather than categories 1 to 3; up to a respondent with *θ* = 1.91 has a 50% chance of choosing category 5 rather than categories 1 to 4. The results also showed differences in difficulty between categories on the response scale across and within items. For differences across items, this means that the respondent has to attain different value of latent trait to select a particular category on the response scale. For example, for choosing category 5 on the response scale, the respondent has to have a latent trait value of at least 0.79 for item 7 but 1.91 for item 1.

**Fig. 2:**
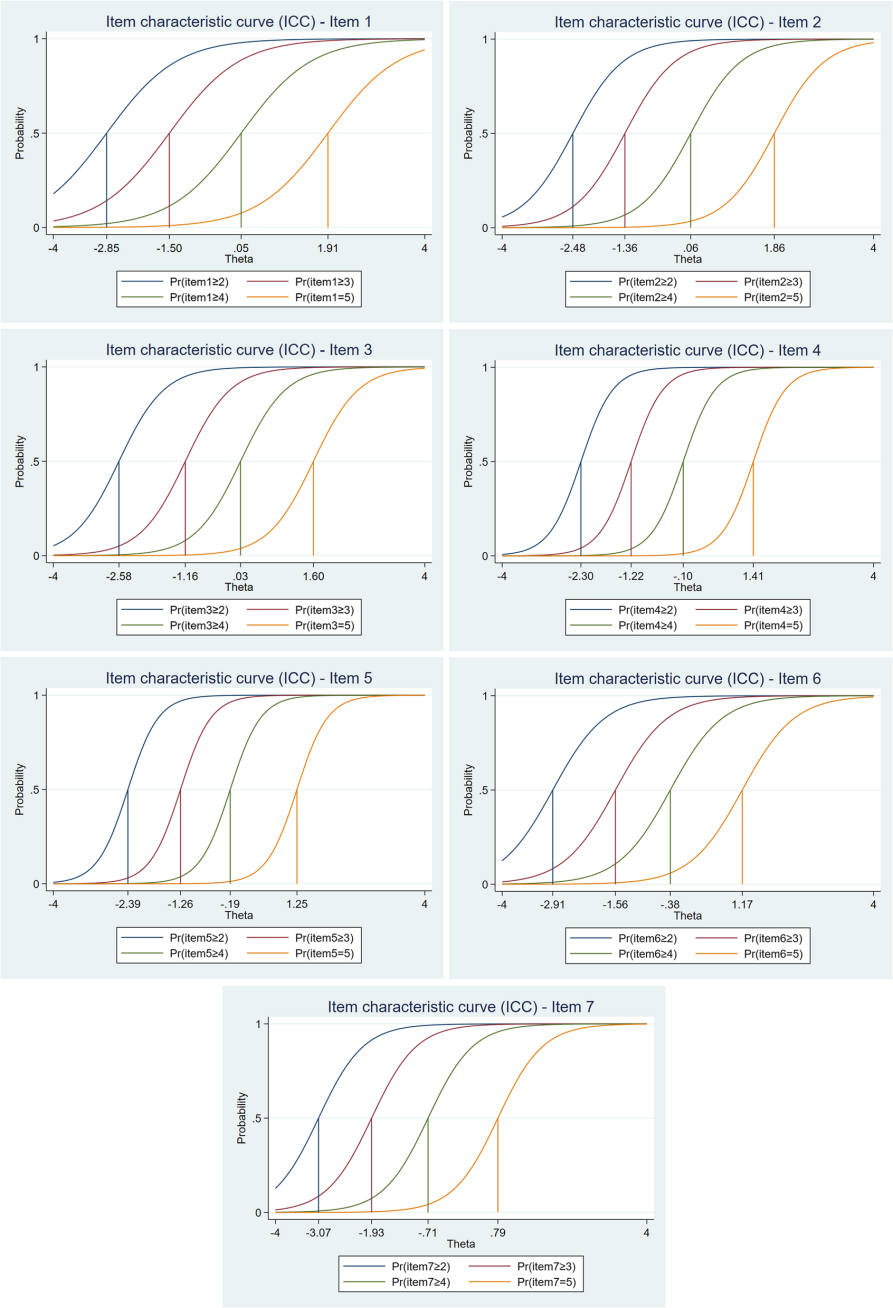
Item characteristic curves (ICCs) for each item of the SWEMWBS

Differences within items are demonstrated by unequal distance between categories on the response scale. For example, for item 2, the difference between thresholds *b1* and *b2* is − 1.12, between *b2* and *b3* is − 1.42, and between *b3* and *b4* − 1.86. From this perspective, item 5 showed the best functioning with differences of − 1.13, − 1.07, and − 1.43. The results described above are also supported graphically through ICCs (see Fig. 2).

**Fig. 3:**
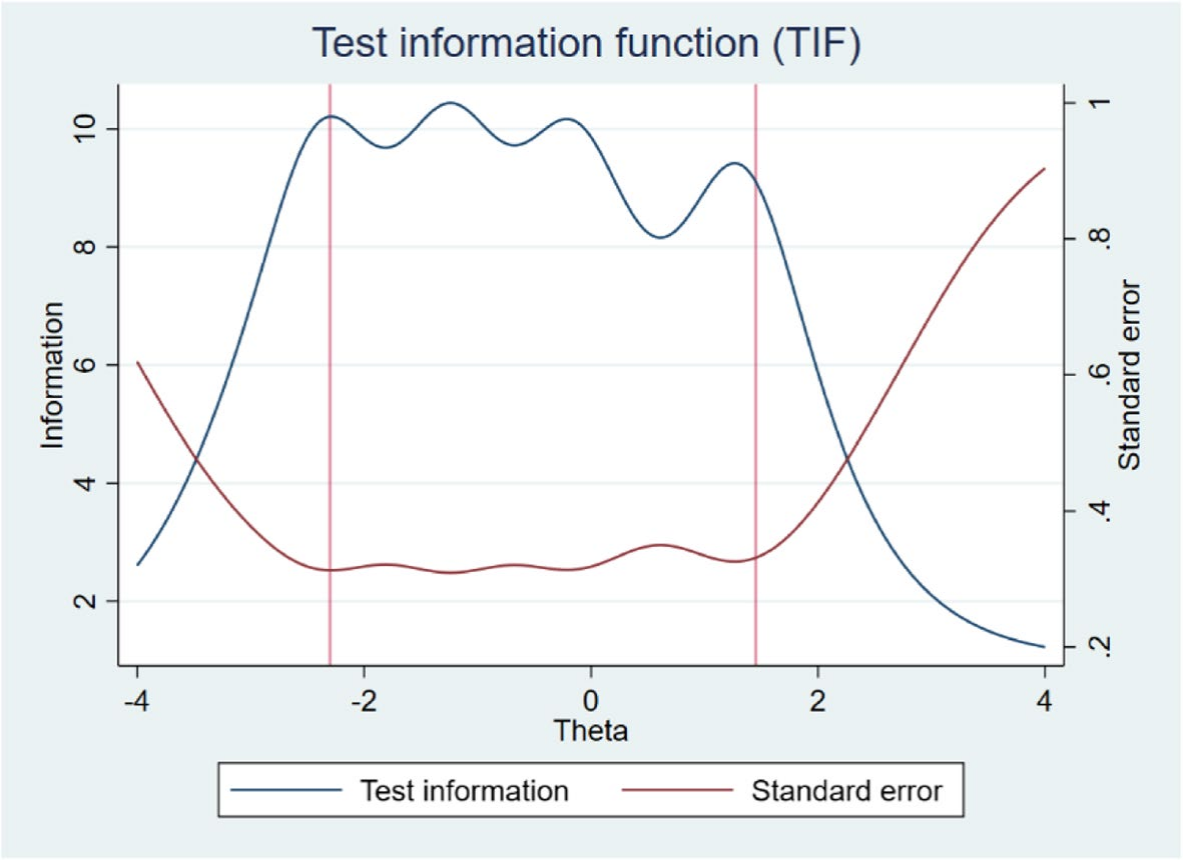
Test information function (TIF) and Standard Error for SWEMWBS

The functioning of the scale as a whole is shown in Fig. 3. The TIF indicates that the scale functions very well, especially between − 2.30 to 1.40 of the latent trait continuum, a range within which the standard error is also smallest. This figure also illustrates the previously presented finding that the SWEMWBS performs better on the left side, i.e., at negative values of the latent trait.

**Fig. 4:**
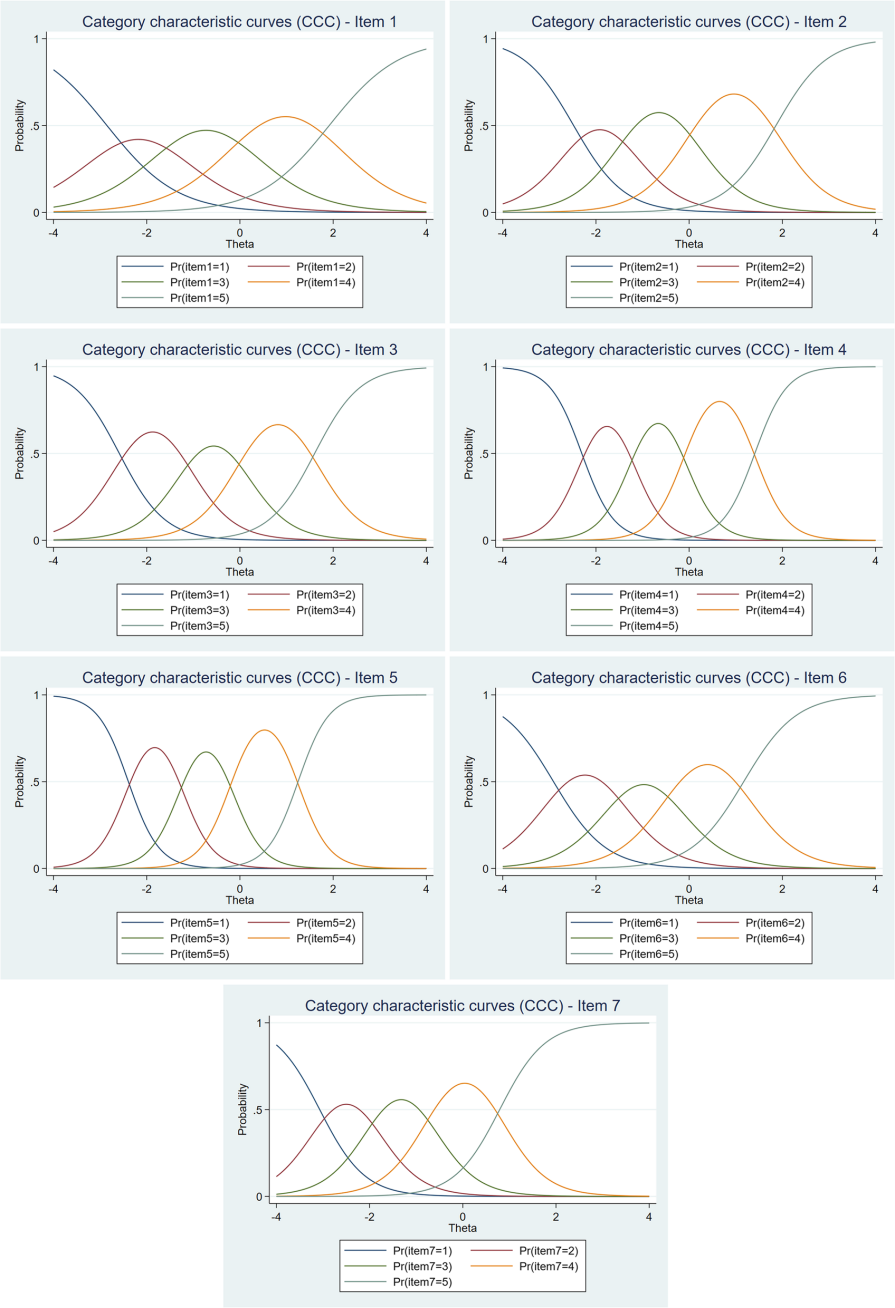
Category characteristic curves (CCCs) for each item of the SWEMWBS

The last part of the IRT analysis focused on evaluating the functioning of the response scale based on CCC for each item from SWEMWBS (see Fig. 4). These curves show how well or poorly each response category performs both in the context of the whole scale and especially when moving between adjacent categories. Ideally, each category should be the most probable in some part of the latent trait, meaning it should have a clear peak and not be overlapped by another category along its entire length. A related issue is that each higher category should be selected with a higher probability than a lower category as the value of the latent trait increases [22]. The slope (parameter *a*) and location (parameter *b*) of the curves are also important for interpretation. Specifically, the greater the slope, the higher the peak of the curve, and the probability of selecting a different category changes more rapidly along the latent trait continuum *θ* [23]. The results show that the response scales for all items of the SWEMWBS show good functioning and that the number of response scale categories is adequate. For the CCCs of all items, each category has a clear peak, indicating that respondents are able to distinguish well between all five categories and use them appropriately. In terms of the shape of the curve, items 4 and 5 perform best since their slope is steeper, whereas item 1 performs slightly worse in this respect.

### Reliability and criterion-related validity

Reliability was tested using Cronbach’s alpha (*α*) and McDonald’s omega (*ω*) and was estimated 0.858 for *α* and 0.857 for *ω*. To assess the criterion validity of the SWEMWBS, the correlation with other relevant measures was calculated. The results shown in Table 3 indicated a very good criterion validity of the SWEMWBS as correlation with all measures can be considered to be large (*r* >  = 0.30) by the criterion of Gignac and Szodorai [24]. The largest negative correlation (− 0.59) was found with the GHQ-12 scale, which is consistent with previous studies (e.g., [8, 12, 25, 26]), because this instrument measures the opposite of mental well-being, psychological distress. On the contrary, a high positive correlation was found with satisfaction with life overall (0.47). The lowest positive correlation was identified for the subjective assessment of general health, namely 0.31. This positive relationship has also been supported in other studies (e.g., [12, 26]). It should be noted, however, that although the correlation is high, these are not identical concepts and it is necessary to distinguish between mental well-being and other concepts such as life satisfaction, subjective health, positive and negative emotions, distress, etc., as these are usually only partial components of well-being.

**Table 3:** Correlations of the SWEMWBS with other relevant measures

	*r*	CI 95%
GHQ-12	− .59^**^	[− .60; − .57]
Life satisfaction	.47^**^	[.46; .49]
General health^a^	.31^**^	[.29; .33]

Pearson correlation coefficient

^**^p < .01

^a^The original response scale has been reversed so that a higher value means better health

## Discussion

The SWEMWBS is one of the most widely used instruments for measuring mental well-being. Although this scale, and especially the original 14-item version (WEMWBS), has been and still is frequently tested for different populations (e.g.; [10, 25, 27, 28]) and validated in different languages (e.g.; [13, 26, 29–33]), this study can be considered innovative mainly for two reasons. First, it is the first study to test the psychometric properties of the SWEMWBS through IRT on a large research sample, which is essential for the use of IRT. To date, IRT has only been used once to test the original 14-item WEMWBS, but on a small sample of the adult population [34]. Second, it focuses on a narrow group of adolescents aged 16 to 19 years, which can be identified as the most vulnerable in terms of susceptibility to mental problems. Most of previous studies have focused either on early adolescents (12 to 15 years) or on the adult population only (e.g.; [25, 26, 35–37]).

First, the assumptions for the use of IRT were verified. Unidimensionality was tested through PCA, which clearly confirmed that SWEMWBS consists of items forming a single factor. To test local independence, residual correlations between pairs of items were computed. Although, in a few cases, residual correlations were slightly above the threshold value (> 0.20) [21], the assumption of local independence was considered to be satisfied due to the quality of the data used and the positive wording of the items only. Related to this was the confirmation of the monotonicity assumption that selecting a higher category on the response scale indicated a higher level of mental well-being.

Items from SWEMWBS are polytomous, and therefore the GRM was applied and preferred over the GPCM based on the better performance of the three indices of fit (Log-likelihood, AIC, BIC). The results computed from the GRM showed very good discriminative power for all items, which according to Baker [19], can be described as “high” for item 1 (1.33) and “very high” for the remaining items (values ranged from 1.78 for item 6 to 3.04 for item 5). This is related to the information contribute by each item to the scale (see Fig. 1), as the most discriminative items, 4 and 5, can also be described as the most informative, while item 1 is the least informative. Based on the difficulty parameters or thresholds (*b*), of which a total of 28 were estimated, it appeared that negative values predominated and that the scale thus better covered the left (negative) side of the latent trait (mental well-being) continuum. A more detailed analysis of the *b* parameters also showed that there were differences in the distance between response scale categories, both between and within items. Although this is an interesting finding that has not yet been articulated and examined in other studies, in general, the five-category response scale performed very well. The result of the functioning of the response scale was very clearly visible in the CCC (see Fig. 4), in which, for each item, the curve for each category had a clear peak and was the most probable in some parts of the latent trait. This can be described as a desirable result indicating good functioning. In terms of the functioning of the scale as a whole, its very high informational contribution was demonstrated, especially between − 2.30 to 1.40 of the latent trait continuum, where the smallest standard error is also indicated.

Finally, for overall evaluation, the reliability and criterion validity of SWEMWBS were tested. The reliability coefficients alpha (0.858) and omega (0.857) had high values, confirming the strong internal consistency of the SWEMWBS and the appropriateness of all its items. This result is consistent with the findings of previous studies (e.g.; [8, 26, 37]). Three instruments were used to test criterion validity: the GHQ-12 to measure psychological distress, one question on general life satisfaction, and one question on subjective assessment of general health. The resulting correlation coefficients were also consistent with the results of other studies (e.g.; [8, 12, 25, 26]), with a high negative correlation with the GHQ-12 and a high positive correlation with overall life satisfaction and self-reported health.

## Conclusions

The 7-item SWEMWBS is confirmed as a concise, reliable, and valid instrument for measuring mental well-being among older UK adolescents. Psychometric analysis of the SWEMWBS using IRT indicates its good quality both at scale-level and item-level. The number of items and their wording are appropriate. The response scale with five categories works very well, and no adjustments are needed. Criterion validity is supported by a high correlation with other relevant instruments. Based on the results presented in this study, the SWEMWBS can be recommended for reliable measurement of mental well-being in the population of adolescents aged 16 to 19 years.

### Supplementary Information

**Additional file 1: Table A1.** Results of principal component analysis (PCA): total variance explained. **Figure A1.** Scree plot from PCA. **Table A2.** Residual correlation between pairs of SWEMWBS items.

## Data Availability

The data is publicly available for download from the UK Data Service. University of Essex, Institute for Social and Economic Research. Understanding Society: Waves 1-12, 2009-2021 and Harmonised BHPS: Waves 1-18, 1991-2009. [data collection]. 17th Edition. UK Data Service, 2023. Accessed 26 June 2023. 10.5255/UKDA-SN-6614-18.

## Abbreviations

AIC: Akaike information criterion
BIC: Bayesian information criterion
CAPI: Computer Assisted Personal Interviewing
CCC: Category characteristic curve
CI: Confidence interval
CTT: Classical Test Theory
GPCM: General Partial Credit Model
GRM: Graded Response Model
GHQ-12: 12-Item General Health Questionnaire
ICC: Item characteristic curve
IIF: Item information function
IRT: Item Response Theory
M: Mean
PCA: Principal component analysis
SD: Standard deviation
SWEMWBS: Short Warwick‑Edinburgh Mental Well-Being Scale
TIF: Test information function
UK: United Kingdom
WEMWBS: Warwick‑Edinburgh Mental Well-Being Scale
WHO: World Health Organization

## Footnotes

1 If respondent participated in more than one wave, then was retained in the data only once at the first interview. In total, there were 1,357 respondents.

2 SWEMWBS is protected by copyright. For commercial and non-commercial use, you must apply for a license from University of Warwick at this website: https://warwick.ac.uk/fac/sci/med/research/platform/wemwbs/using
