# Supplementary material for: Item response theory-based psychometric analysis of the Short Warwick-Edinburgh Mental Well-Being Scale (SWEMWBS) among adolescents in the UK
Source: Health Qual Life Outcomes. 2023 Sep 29;21:108. doi: 10.1186/s12955-023-02192-0 (PMC10540427; doi:10.1186/s12955-023-02192-0)
Supplement: Supplementary file 1 — Additional file 1: Table A1. Results of principal component analysis (PCA): total variance explained. Figure A1. Scree plot from PCA. Table A2. Residual correlation between pairs of SWEMWBS items. [file 12955_2023_2192_MOESM1_ESM.docx]

**Additional file 1**

**Table A1** Results of principal component analysis (PCA): total variance explained

| Component | Initial Eigenvalues | | | Extraction Sums of Squared Loadings | | |
| --- | --- | --- | --- | --- | --- | --- |
|  | Total | % of Variance | Cumulative % | Total | % of Variance | Cumulative % |
| 1 | 3,820 | 54,567 | 54,567 | 3,820 | 54,567 | 54,567 |
| 2 | ,839 | 11,981 | 66,549 |  |  |  |
| 3 | ,609 | 8,705 | 75,254 |  |  |  |
| 4 | ,525 | 7,497 | 82,750 |  |  |  |
| 5 | ,456 | 6,514 | 89,265 |  |  |  |
| 6 | ,413 | 5,901 | 95,166 |  |  |  |
| 7 | ,338 | 4,834 | 100,000 |  |  |  |
| Extraction Method: Principal Component Analysis. | | | | | | |


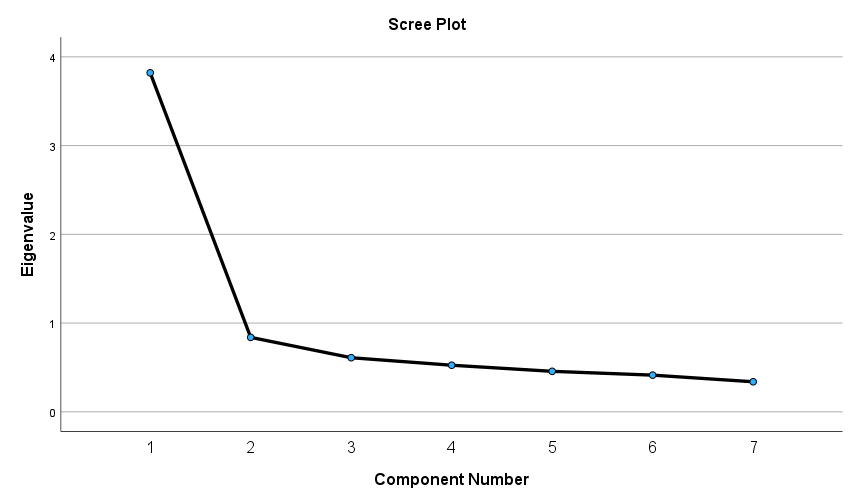


**Figure A1**. Scree plot from PCA

**Table A2** Residual correlation between pairs of SWEMWBS items

|  | item 1 | item 2 | item 3 | item 4 | item 5 | item 6 | item 7 |
| --- | --- | --- | --- | --- | --- | --- | --- |
| item 1 | 1.00 | .21 | –.14 | –.15 | –.24 | –.04 | –.12 |
| item 2 |  | 1.00 | –.09 | –.19 | –.26 | –.07 | –.18 |
| item 3 |  |  | 1.00 | –.14 | –.15 | –.12 | –.22 |
| item 4 |  |  |  | 1.00 | –.22 | –.22 | –.21 |
| item 5 |  |  |  |  | 1.00 | –.22 | –.11 |
| item 6 |  |  |  |  |  | 1.00 | –.03 |
| item 7 |  |  |  |  |  |  | 1.00 |
